# IncobotulinumtoxinA for Upper Limb Essential Tremor: A Randomized, Double-Blind, Placebo-Controlled Trial

**DOI:** 10.3390/toxins18100435

**Published:** 2026-10-09

**Authors:** David M. Simpson, Marta Banach, Jason Aldred, Jack Lee, William Ondo, Fernando Pagan, Anastasia Bagretsova, Gudrun Klein, Thorin L. Geister, Irena Pulte, Mandar Jog

**Affiliations:** 1 Department of Neurology, Icahn School of Medicine at Mount Sinai, New York, NY 10029, USA; 2 Specjalistyczne Gabinety Sp. z o.o.2, plac Lasoty 4, 30-539 Kraków, Poland; 3 Selkirk Neurology, Spokane, WA 99202, USA; jaldred@selkirkneurology.com; 4 MDDT Inc., London, ON N6G 0J3, Canada; jacklee@mddtinc.ca; 5 Houston Methodist Neurological Institute, Houston, TX 77030, USA; 6 Weill Cornell Medical School, Manhattan, New York, NY 10021, USA; 7 Medstar Georgetown Neurology at McLean, McLean, VA 22101, USA; fernando.l.pagan@gunet.georgetown.edu; 8 Merz Therapeutics GmbH, 60318 Frankfurt am Main, Germanyirena.pulte@merz.de (I.P.); 9 University Hospital—London Health Sciences Centre, London, ON N6A 5A5, Canada; mandar.jog@lhsc.on.ca

**Keywords:** essential tremor, upper limb, incobotulinumtoxinA, efficacy, safety

## Abstract

This double-blind, placebo-controlled, phase II trial (NCT04766723) investigated the efficacy and safety of incobotulinumtoxinA (Xeomin^®^) in the treatment of essential tremor (ET) of the upper limb (UL). Adults with ET of the UL were randomized 2:1 (*n* = 78) and treated (*n* = 77) with intramuscular injections of incobotulinumtoxinA or placebo at semi-flexible doses into their motor-dominant arm. The primary endpoint was the change from baseline to week 6 in maximum tremor amplitude at wrist level. Baseline characteristics of treated patients were well matched across treatment arms. The primary endpoint was not met; maximum tremor amplitude at the wrist decreased from baseline to week 6 in both groups, with changes numerically favoring placebo (least squares mean treatment difference 0.21, *p* = 0.28). At week 6, compared with the placebo group, the incobotulinumtoxinA group had improvement in The Essential Tremor Rating Assessment Scale (TETRAS) performance dominant UL score (*p* = 0.006) and Quality of Life in ET (QUEST) total score (*p* = 0.073). However, these secondary outcomes must be interpreted descriptively as exploratory findings. Although the *p*-value and the 95% confidence interval including zero did not support statistical significance, the observed numerical treatment difference in change from baseline in QUEST total score suggests that the treatment effect may be consistent with the minimal clinically important difference in favor of incobotulinumtoxinA. Improvement in TETRAS Activities of Daily Living UL score was similar in both groups at week 6 (*p* = 0.933). IncobotulinumtoxinA was generally well tolerated. Treatment of UL ET with incobotulinumtoxinA did not show superiority over placebo for the primary endpoint. Secondary and other efficacy endpoints should therefore be viewed as exploratory, generating hypotheses suggesting potential improvements in TETRAS UL performance score and quality of life that require confirmation in further research. IncobotulinumtoxinA demonstrated a favorable safety profile without any new or unexpected safety concerns.

## 1. Introduction

Tremor is defined as an involuntary rhythmic oscillation of a part of the body [1]. The most recent consensus statement from the task force on tremor of the International Parkinson and Movement Disorder Society defines patients with essential tremor (ET) as those with an isolated syndrome of bilateral upper limb (UL) action tremor of at least 3 years duration, with or without tremor in other locations (e.g., head, voice, or lower limbs), and the absence of other neurological signs, such as dystonia, ataxia, or parkinsonism [1]. ET is one of the most common movement disorders and the most prevalent form of tremor [2]. ET is a chronic, progressive neurological condition with an overall estimated prevalence of 1.33% [3]. The incidence of ET increases with advancing age in both males and females [3]. The severity of ET and its associated disability and burden varies greatly. Mild tremor is associated with limited disability, whereas a severe bilateral tremor can significantly impede patients from performing activities of daily living (ADL) [4,5]. ET can also adversely affect mental health and quality of life [6].

Current non-surgical therapeutic options for ET are limited. Standard drug treatments such as oral propranolol and primidone are associated with a considerable rate of side effects, frequently leading to withdrawal of treatment [7]. Botulinum neurotoxin type A (BoNT A) has been investigated clinically for limb tremor in ET. Initial double-blind, placebo-controlled studies used fixed doses of onabotulinumtoxinA and reported efficacy over placebo in patients with ET of the hand [8,9]. However, both studies identified an increased risk of hand or finger weakness after onabotulinumtoxinA treatment.

Subsequent open-label studies suggested clinical benefit from baseline and improved tolerability when onabotulinumtoxinA was administered at customized dosages to only the wrist flexor muscles of patients with ET, based on landmark-guided injections [10,11]. Other small, open-label studies of customized dosing using incobotulinumtoxinA (IncoBoNT-A, Xeomin^®^), a botulinum neurotoxin type A product free from complexing proteins [12], demonstrated good tolerability and improvement in clinical and kinematic assessments of ET [13,14]. They emphasized the importance of injecting the shoulder and elbow muscles and advocated for the use of kinematics-guided dosing to optimize treatment of ET [13,14].

An exploratory, double-blind, placebo-controlled, phase II pilot study (NCT02207946) (*n* = 30) investigated the safety and efficacy of unilateral treatment with IncoBoNT-A for the treatment of ET of the UL over 24 weeks [15]. IncoBoNT-A had a favorable safety profile and demonstrated superiority over placebo in both tremor amplitude at the wrist (reduction from study baseline to week 4) and physician’s global impression of change scale (GICS; improvement from study baseline to week 4 compared with placebo).

A systematic review of 12 studies evaluating BoNT A treatment for UL ET concluded that there were improvements in tremor severity scores, motor function, and quality of life. Moreover, BoNT A injections exhibited a favorable safety profile, supporting their potential as a viable therapeutic alternative for UL ET based on the positive risk–benefit assessment [16]. However, muscle weakness was reported, which is a recurrent concern and is central to clinical translation.

The current proof-of-concept phase II study built on experience from previous BoNT A studies, particularly the double-blind pilot study of IncoBoNT-A [15]. Its aim was to generate high-quality data as the foundation of a more evidence-based approach to BoNT A treatment of ET of the UL. The primary endpoint in the current study was informed by learnings from the previous pilot study. In that study, the log-transformed accelerometric hand-tremor amplitude measured by kinematic tremor analysis was the endpoint most sensitive to change. The selection also reflected the established contribution of wrist muscles to tremor amplitude and the clinical relevance of wrist-level tremor, which is known to substantially interfere with ADL.

## 2. Results

### 2.1. Patient Demographics, Disposition, and Extent of Exposure

Patients were enrolled between 24 February 2021 and 15 March 2023 by investigators at 14 study sites in the USA, Canada, and Poland. Of these, 12 sites (USA: 7, Canada: 1, Poland: 4) randomized at least one patient. A total of 38 patients were randomized in the USA, 5 in Canada, and 35 in Poland.

A total of 78 patients were randomized: 51 patients to IncoBoNT-A (of whom all were treated) and 27 to placebo (of whom 26 were treated). However, one patient randomized to IncoBoNT-A was erroneously treated with placebo. Accordingly, in cycle 1, the number of patients treated with IncoBoNT-A was 50, and the number treated with placebo was 27.

**Figure 1:**
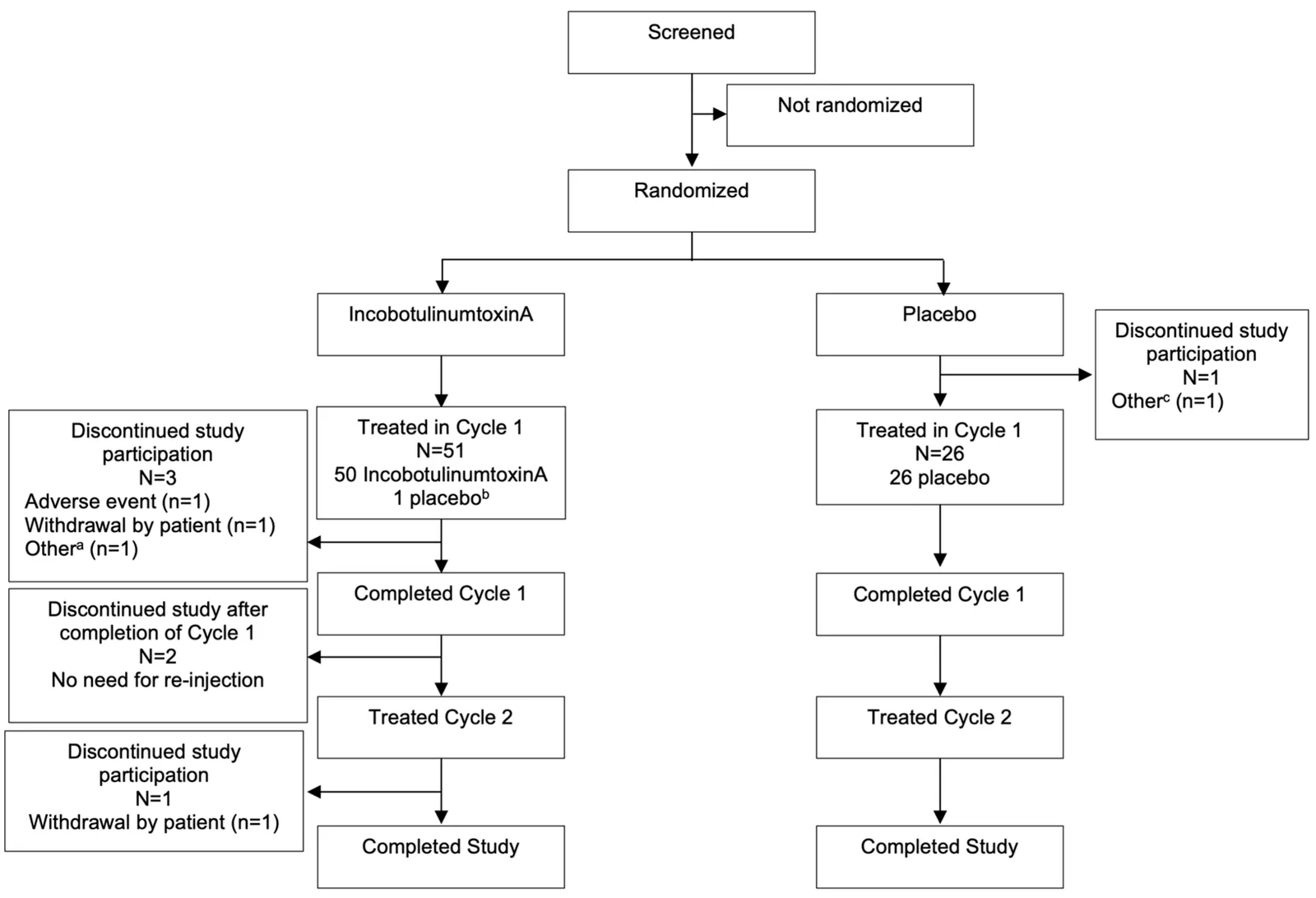
Patient disposition. ^a^ Patient did not wish to continue but did not withdraw consent; ^b^ Patient received placebo in error; ^c^ Patient was randomized in error but did not receive an injection. The safety evaluation set (SES) for the unilateral treatment period (SES-UP) included 77 patients (incobotulinumtoxinA: 50; placebo: 27); the SES for the bilateral treatment period (SES-BP) included 72 patients (incobotulinumtoxinA: 45; placebo: 27); the full analysis set (FAS) for the unilateral treatment period (FAS-UP) included 76 patients (incobotulinumtoxinA: 50; placebo: 26); and the FAS for the bilateral treatment period (FAS-BP) included 64 patients (incobotulinumtoxinA: 42; placebo: 22).

In cycle 1, three patients in the IncoBoNT-A group discontinued for the following reasons: adverse event (AE) unrelated to the treatment (*n* = 1), withdrawal from the study (*n* = 1), and “other” (*n* = 1). One patient in the placebo group discontinued (reason: “other”). The CONSORT flow diagram of study patients provides full details (Figure 1). The study had a high completion rate: 94.9% of all randomized patients completed cycle 1 and 91.0% the entire study.

Patient demographics and baseline characteristics of ET were broadly similar in the two groups at baseline (Table 1). Overall, 53.2% of patients were male, the mean ± standard deviation (SD) age was 65.1 ± 11.6 years, and 98.7% of patients were white. The mean ± SD age of ET onset was 39.7 ± 18.8 years, and ET duration was 25.4 ± 17.3 years. The majority (61.0%) of patients had a family history of ET.

Patients in the IncoBoNT-A group had a slightly lower mean maximum tremor amplitude at wrist level of their motor-dominant UL at baseline but were otherwise comparable with regard to baseline status of efficacy variables. The small between-group difference in mean QUEST total score was negligible given the large standard deviations in each group (Table 1).

Concomitant medication usage was overall comparable between the IncoBoNT-A and placebo groups (Table 1). Differences between the treatment groups were smaller than 10% with respect to medications frequently used for the treatment of ET (i.e., propranolol, primidone, and topiramate). Data on extent of exposure, including the numbers and proportions of participants injected per muscle and muscle group, as well as mean (SD) injection volume for each muscle, muscle group, and UL, indicated that the planned injection scheme (Table S3) was generally well adhered to during both the unilateral treatment period for the treated UL (Table S4) and the bilateral treatment period for both ULs (Table S5).

### 2.2. Efficacy Endpoints

#### 2.2.1. Primary Efficacy Endpoint

**Figure 2:**
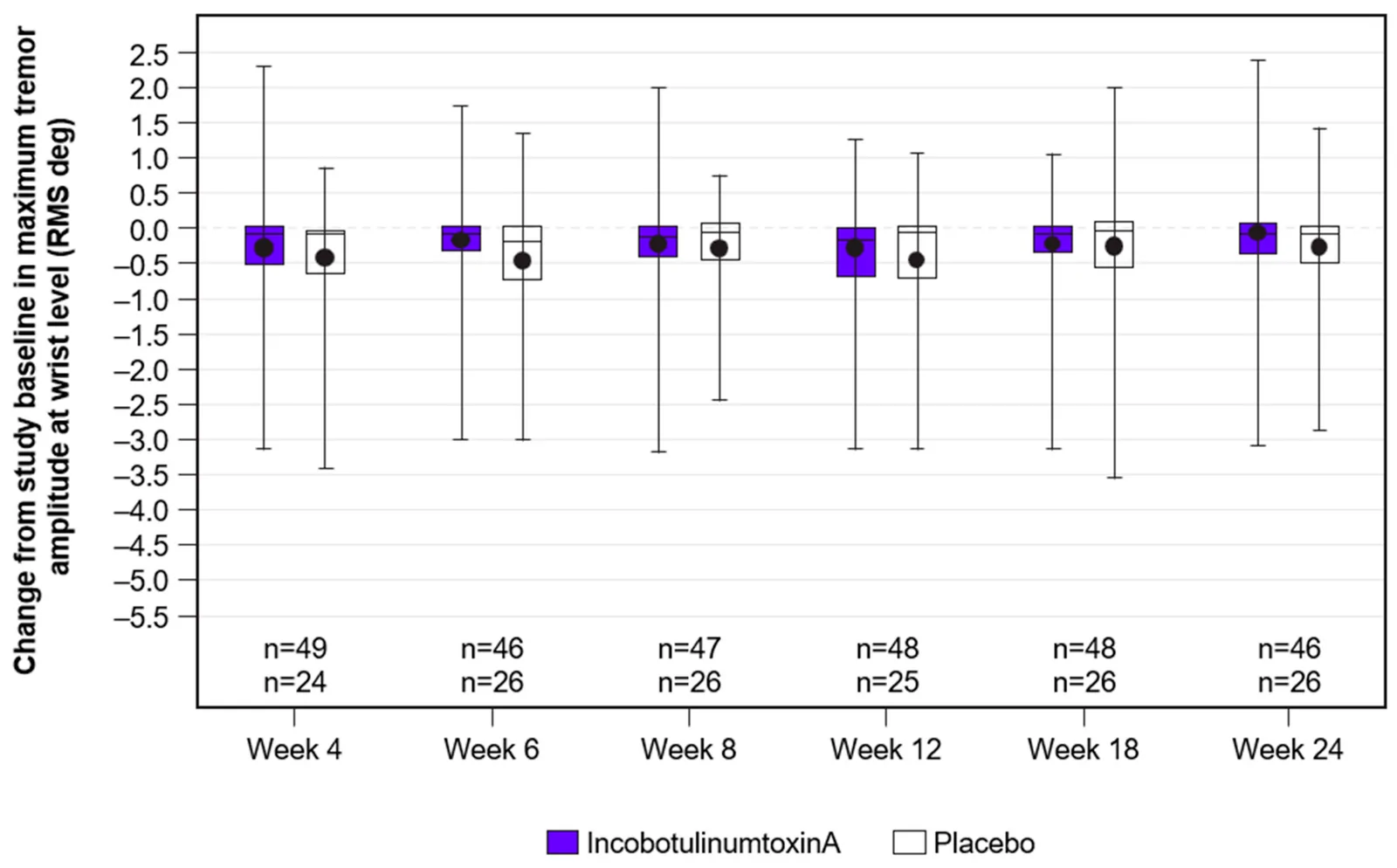
Time course of the change from study baseline in the maximum tremor amplitude at wrist level (full analysis set for the unilateral treatment period). Lower and upper whiskers represent minimum and maximum values, boxes represent quartiles 1 and 3, horizontal lines within the boxes show median values, and the filled circles show mean values. Changes < 0 indicate improvements. deg, degrees of arc; RMS, root mean square.

At study baseline, root mean square (RMS) ± SD maximum tremor amplitude at wrist level was 0.98 ± 1.12 degrees in the IncoBoNT-A and 1.16 ± 1.18 degrees in the placebo group. At week 6, the least squares means (LSM) (95% confidence interval [CI]) change from baseline (primary endpoint) from the baseline-adjusted analysis of covariance (ANCOVA) was −0.25 (−0.53–0.04) in the IncoBoNT-A group and −0.46 (−0.83 to −0.09) in the placebo group. The LSM treatment difference (95% CI) of 0.21 (−0.18–0.61) did not reach statistical significance (*p* = 0.28), and the primary endpoint was not met. The tremor amplitude numerically reduced from baseline in both treatment groups; reductions were seen as early as week 4 and remained stable over time for both groups until week 18 of the unilateral treatment period (Figure 2).

#### 2.2.2. Key Secondary Endpoints

**Figure 3:**
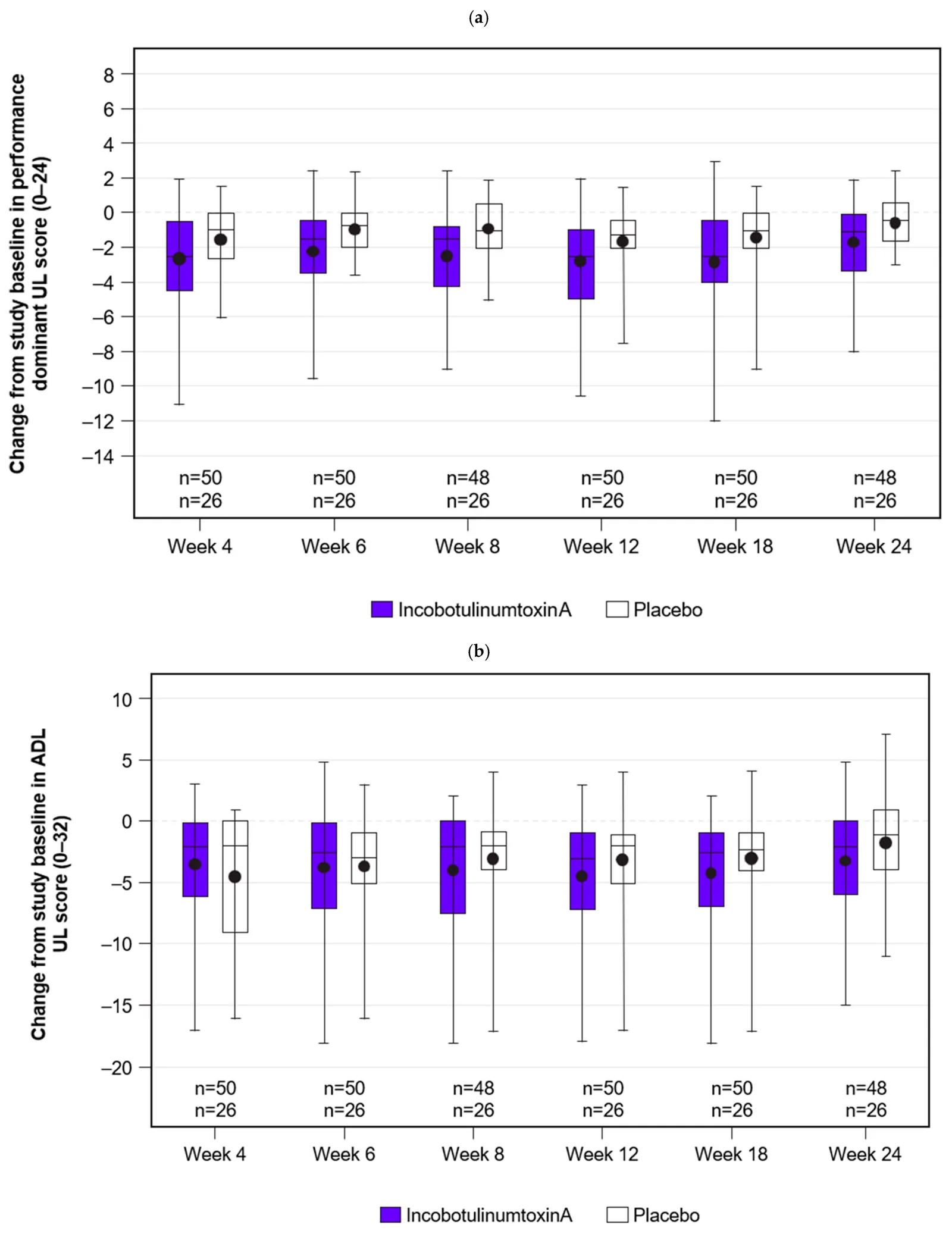
Time course of the change from study baseline in (**a**) TETRAS performance dominant UL and (**b**) TETRAS ADL UL scores during the unilateral treatment. Lower and upper whiskers represent minimum and maximum values, boxes represent quartiles 1 and 3, horizontal lines within the boxes show median values and the filled circles show mean values. Changes < 0 indicate improvements. ADL, activities of daily living; TETRAS, The Essential Tremor Rating Assessment Scale; UL, upper limb.

The Essential Tremor Rating Assessment Scale (TETRAS) performance dominant UL scores (mean ± SD) at baseline were 13.0 ± 3.0 in the IncoBoNT-A group and 12.3 ± 2.1 in the placebo group. The LSM (95% CI) change from baseline to week 6 was −1.98 (−2.79 to −1.18) for the IncoBoNT-A group and −0.69 (−1.47–0.09) for the placebo group. The LSM treatment difference (95% CI) of −1.29 (−2.19 to −0.39) (*p* = 0.006) favored IncoBoNT-A. In addition, mean reductions from study baseline in TETRAS performance dominant UL score were numerically greater for the IncoBoNT-A group than for the placebo group at all post-baseline visits of the unilateral treatment period, i.e., until week 24, peaking at week 12 (Figure 3a).

At study baseline, the mean ± SD TETRAS ADL UL score was 19.4 ± 4.0 in the IncoBoNT-A group and 19.7 ± 3.0 in the placebo group. At week 6, the LSM (95% CI) change in TETRAS ADL UL score (key secondary endpoint) was −3.41 (−5.09 to −1.73) in the IncoBoNT-A group and −3.32 (−5.39 to −1.24) in the placebo group. The LSM (95% CI) treatment difference was −0.09 (−2.34–2.15) (*p* = 0.933). Over the course of the unilateral treatment period, improvements in mean TETRAS ADL UL scores from baseline were observed for both groups (Figure 3b).

#### 2.2.3. Other Secondary and Other Important EFFICACY Endpoints for the Unilateral Treatment Period

**Figure 4:**
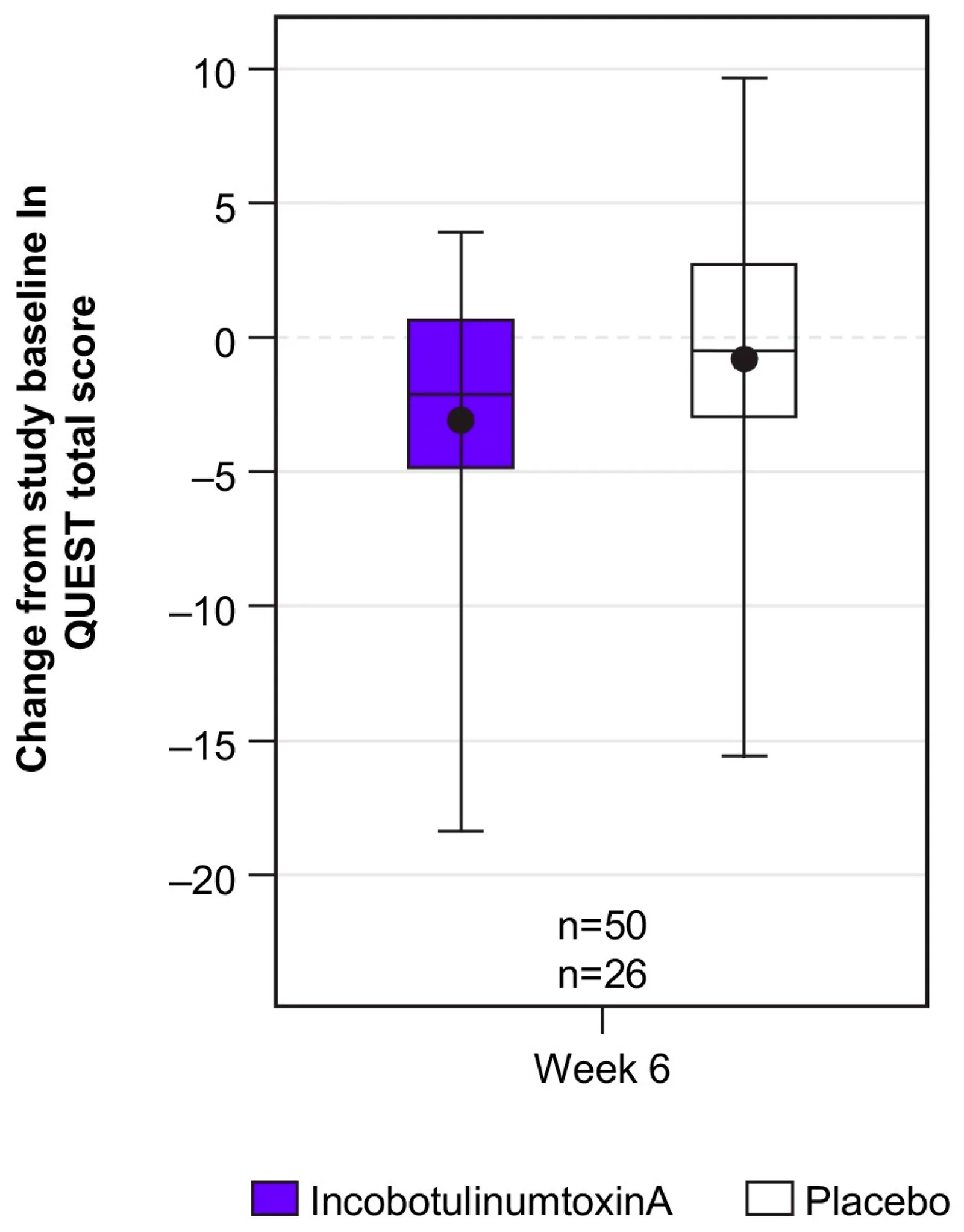
Change from study baseline to week 6 in QUEST total score during the unilateral treatment period. Lower and upper whiskers represent minimum and maximum values, boxes represent quartiles 1 and 3, horizontal lines within the boxes show median values, and the filled circles show mean values. Changes < 0 indicate improvements. QUEST, Quality of Life in Essential Tremor Questionnaire.

At study baseline, mean ± SD Quality of Life in Essential Tremor Scale (QUEST) total score was 33.8 ± 16.2 in the IncoBoNT-A group and 30.7 ± 15.5 in the placebo group. At week 6, mean reductions from study baseline were numerically greater (indicating larger improvements in ET-related quality of life) in the IncoBoNT-A group than in the placebo group (Figure 4). The IncoBoNT-A group showed an LSM (95% CI) reduction in QUEST total score of −5.49 (−8.74 to −2.24) compared with −1.01 (−5.70–3.68) for the placebo group. The LSM (95% CI) treatment difference was −4.48 (−9.39–0.44) (*p* = 0.073). Although the *p*-value, and that the 95% CI included zero, did not support statistical significance, the observed numerical treatment difference in change from baseline in QUEST total score suggested that the treatment effect may have met the minimal clinically important difference for the QUEST in favor of the IncoBoNT-A group [17].

At study baseline, the mean ± SD TETRAS ADL functional impact score was 7.5 ± 1.6 in the IncoBoNT-A group and 7.3 ± 1.4 in the placebo group. The LSM (95% CI) reduction from baseline to week 6 was comparable between the IncoBoNT-A (−1.11 [−1.71 to −0.51]) and the placebo groups (−1.20 [−2.06 to −0.34]). The LSM treatment difference (95% CI) of 0.09 (−0.81–1.00) did not reach statistical significance (*p* = 0.83).

For the IncoBoNT-A group, mean ± SD GICS scores of the motor-dominant UL at week 6 assessments were 0.9 ± 1.3 according to patients’ assessments and 1.2 ± 1.4 according to investigators’ assessments, which showed numerically better improvements than for the placebo group: 0.8 ± 1.1 according to patients’ assessments and 0.8 ± 1.0 according to investigators’ assessments. No statistical difference in LSM treatment differences (95% CI) from the baseline-adjusted ANCOVA comparing IncoBoNT-A to placebo was recorded at week 6 in either the patients’ GICS score (0.19 [−0.35–0.73]; *p* = 0.486) or the investigators’ GICS score (0.44 [−0.13–1.01]; *p* = 0.126).

#### 2.2.4. Secondary and Other Important Efficacy Endpoints for the Bilateral Open-Label Treatment Period

All TETRAS scores reduced from cycle 2 baseline (C2BL) to week 6 of the bilateral open-label IncoBoNT-A treatment period. For the total full analysis set for the bilateral treatment period (FAS-BP), mean ± SD TETRAS performance dominant UL score was 11.5 ± 2.81 at C2BL, and change from C2BL to week 6 was −2.8 ± 2.62. Mean ± SD TETRAS performance subscale score was 26.6 ± 6.74 at C2BL, and change from C2BL to week 6 was −5.6 ± 5.14. At C2BL, mean ± SD TETRAS ADL UL score was 17.2 ± 5.34, and change from C2BL to week 6 was −4.4 ± 4.07. TETRAS ADL functional impact score was 6.5 ± 1.75 at C2BL. Change from C2BL to week 6 was −1.4 ± 1.42.

Maximum angular tremor amplitude at wrist level also reduced from C2BL to week 6 of the bilateral open-label IncoBoNT-A treatment period. The RMS ± SD was 0.93 ± 1.40 degrees at C2BL, and change from C2BL baseline to week 6 was −0.43 ± 1.19 degrees.

Mean ± SD QUEST total score was 27.0 ± 16.14 at C2BL. Change from C2BL baseline to week 6 was −4.95 ± 8.30.

### 2.3. Safety Assessment and Endpoints

A summary of treatment-emergent adverse events (TEAEs) in the unilateral treatment period is provided in Table 2. A larger proportion of patients in the IncoBoNT-A group than in the placebo group reported one or more TEAE related to treatment (30.0% vs. 3.7%). Similarly, reporting of one or more TEAE (60.0% vs. 22.2%), one or more TEAE of special interest (TEAESI [events indicating potential toxin spread]; 26.0% vs. 3.7%), and one or more TEAESI related to treatment (24.0% vs. 0.0%) was more frequent in the IncoBoNT-A group. No severe treatment-related TEAEs occurred. The incidence of treatment-emergent serious adverse events (TESAEs) was similar between the IncoBoNT-A group (6.0%) and the placebo group (7.4%); no TESAE was considered related to treatment.

A summary of TEAEs in the bilateral treatment period is provided in Table 3. Overall, the incidence of any TEAE reported during the 12-week observation period of cycle 2 was lower than during the 24-week cycle 1 among IncoBoNT-A-treated patients. Overall, the incidence of TEAEs related to treatment, TEAESIs, TEAESIs related to treatment, and TESAEs was comparable between cycle 1 and cycle 2 among IncoBoNT-A-treated patients; none of the TESAEs were deemed related to treatment. No fatal TEAEs or TEAEs leading to premature study discontinuation occurred in either the unilateral or the bilateral treatment period.

The most common TEAE during cycle 1 was the TEAESI muscular weakness in the injected UL, in most cases to single fingers or the hand, which was observed exclusively in IncoBoNT-A-treated patients (Table 2). Similar data were reported in the bilateral treatment period (Table 3). In cycle 1, weakness was recorded as mild in 10 (20%) and as moderate in two (4%) of 50 IncoBoNT-A-treated patients. In cycle 2, the results were similar (mild 16/72 [22.2%], moderate 1/72 [1.4%]). No patients reported the intensity of muscular weakness as severe at any time during the study.

In cycle 1, all reported muscular weakness events had recovered/resolved by the end of the study. The median duration was 43 days (range: 12–208 days).

In cycle 2, one patient reported a muscular weakness event that had not recovered/not resolved by the end of the study, and six patients reported muscular weakness events that were recovering/resolving at the end of the study; the duration of these events was unknown. Among the remaining 10 participants whose muscular weakness events had recovered/resolved by the end of the study, the median duration was 67 days (range: 9–81 days).

A post hoc subgroup analysis of patients who reported vs. did not report muscular weakness following IncoBoNT-A treatment in cycle 1 indicated that muscular weakness did not impact improvement in the TETRAS performance dominant UL score at week 6. The mean ± SD change from baseline was −2.50 ± 2.51 among the 12 patients with muscular weakness, compared with −2.21 ± 2.66 among the 38 patients without muscular weakness. Improvement in the TETRAS ADL UL score at week 6 appeared numerically greater in IncoBoNT-A-treated patients with vs. without muscular weakness (mean ± SD change from baseline: −5.58 ± 6.01 vs. −3.16 ± 4.57). However, the large SDs indicate substantial interindividual variability within both subgroups. For patients treated with IncoBoNT-A, mean handheld dynamometer-measured maximum grip strength of the injected UL decreased by 11.1% from study baseline to week 4, and then slowly recovered almost to the starting level by week 24 of the unilateral treatment period, whereas a slight improvement was observed for patients treated with placebo (Table S1). In the non-injected UL, mean grip strength remained largely unchanged from study baseline.

At the end of the study, after bilateral treatment of all eligible patients with IncoBoNT-A, the investigators’ global assessment of tolerability was either “very good” or “good” for 68 of the 71 patients assessed (95.8%). The remaining three patients tolerated the treatment moderately well, according to the investigator.

In summary, no new safety signals were identified, but treatment-related muscular weakness was common and occurred only in patients treated with IncoBoNT-A.

## 3. Discussion

The aim of this proof-of-concept phase II clinical study was to investigate the efficacy, safety, and tolerability of IncoBoNT-A in the treatment of patients with ET of the UL. Although both ULs are involved in ET, tremor affecting the motor-dominant UL is more likely to cause functional disability and impact quality of life [18]. Therefore, during the unilateral treatment period, IncoBoNT-A or placebo was injected into the motor-dominant UL. Bilateral treatment of all patients during the open-label extension period enabled evaluation of IncoBoNT-A in the broader clinical context of ET affecting both ULs. The study population may be considered representative of the broader ET population with respect to mean age, sex distribution, and disease characteristics. The baseline characteristics of the current cohort were generally consistent with those reported in a large meta-analysis of 42 studies examining the ET population worldwide [3]. The primary efficacy endpoint was not met. There was no statistically significant difference between treatment groups in the maximum tremor amplitude at the wrist at week 6, as measured by the TremorTek^®^ device. Indeed, the numerical trend favored placebo for reduction in tremor amplitude (LSM treatment difference 0.21, *p* = 0.28), despite favorable effects of IncoBoNT-A measured using TremorTek^®^ in a prior study [15]. Nevertheless, tremor amplitude decreased from baseline in both treatment groups, and improvements were also observed in both ULs following open-label bilateral treatment with IncoBoNT-A in the second injection cycle. The reduction in tremor amplitude from baseline also observed following placebo treatment in cycle 1 may potentially reflect a practice or habituation effect associated with serial TremorTek assessment sessions. As patients performed four standardized tasks across three recording rounds at each visit during both treatment cycles, increasing familiarity with the assessment procedure over time may have contributed to the observed reduction.

Because device-based tremor measurements obtained at a defined timepoint may not fully reflect tremor-related functional impairment or the clinical relevance of changes in tremor amplitude, clinical measures of functional impact and health-related quality of life, including TETRAS and QUEST, were also assessed. As the primary endpoint was not met and no multiplicity adjustment was applied, findings for these clinical measures were analyzed descriptively and should be interpreted cautiously.

TETRAS performance of the dominant UL suggested improvement from baseline with IncoBoNT-A compared to placebo at week 6, which persisted throughout the 24-week unilateral treatment period. TETRAS performance of the dominant UL also improved and appeared stable over time during the open-label treatment period. One possible reason why TETRAS performance of the dominant UL score may have detected potential between-group differences, whereas TremorTek^®^ did not, is that TETRAS includes a more relevant and complex range of tasks to detect treatment differences (postural tremor [arms outstretched], kinetic tremor [finger-to-nose maneuver], task-specific tremor [Archimedes spiral drawing and handwriting], and dot approximation), whereas TremorTek^®^ assesses four static arm postures. This potential explanation should be considered hypothesis-generating and taken into account when designing future research. The ADL patient-reported outcomes section of TETRAS assesses the occupational impairment and social impact of tremor on daily activities. From baseline to week 6, improvement in TETRAS ADL UL was observed in both treatment groups without a statistically significant between-group difference. At weeks 8 and 12, descriptive analyses showed numerical differences favoring IncoBoNT-A. These observations are exploratory and may inform future hypotheses about the time course of potential treatment effects on daily functioning. We speculate that the beneficial effects of botulinum neurotoxin treatment on daily functioning might become more apparent over time, as patients adapt their daily activities to changes in tremor, whereas standardized performance-based assessments may capture more immediate effects. For QUEST, the treatment differences in the score did not reach statistical significance versus placebo in cycle 1 (as shown by the *p*-value and that the 95% CI for the difference crossed zero). Nevertheless, the observed numerical difference was of a magnitude that may be consistent with a potential clinically important difference [17]. Together with the TETRAS ADL findings, this may suggest that patients require time to test, learn, and perceive changes in their individual living situations and health-related quality of life. However, in the absence of formal responder analyses and given that multiple endpoints were evaluated without multiplicity adjustment, these findings should be regarded as exploratory and hypothesis-generating rather than evidence of established clinical benefit at the individual-patient level. In the phase II pilot study by Jog et al. [15], IncoBoNT-A was superior to placebo for the primary endpoint of reduction in tremor amplitude at the wrist level in patients with ET of the UL. Hand motor function improved after a single injection cycle with IncoBoNT-A versus placebo, as did maximum angular tremor amplitude, Fahn-Tolosa-Marin (FTM) Part B motor performance, FTM ADL, and GICS. QUEST did not improve. Persistent tremor reductions were observed for up to 24 weeks after IncoBoNT-A injections in the pilot study.

Key methodological differences between the pilot study by Jog et al. and the current trial may have contributed to the divergent outcomes. In the pilot study, an individualized IncoBoNT-A treatment approach determined using the TremorTek^®^ device (MDDT Inc., London, ON, Canada) was applied, whereas the current phase II trial used a semi-flexible standardized injection scheme. Samotus et al. [14] also investigated muscle selection and dosing guided by the same computer-assisted kinematic tremor analysis algorithm, thereby reducing clinician subjectivity. Their findings demonstrated that both the wrist and proximal joints contribute to overall tremor amplitude and functional impairment.

Building on these insights, the current study adopted a semi-flexible injection scheme. Fixed doses were administered to predefined shoulder muscles (M. latissimus dorsi, M. pectoralis major, M. supraspinatus, M. infraspinatus) and elbow muscles (M. brachialis and M. triceps brachii), while wrist muscle selection and dosing remained flexible. However, in routine clinical practice, clinicians often prioritize wrist muscle targeting, as injections into shoulder and elbow muscles are perceived to have limited impact on functional improvement. Consequently, future research may benefit from a more rigid, algorithm-driven dosing strategy for wrist muscles, tailored to the individual tremor characteristics of each patient to optimize outcomes.

Recent literature has highlighted increasing placebo response rates in clinical trials [19,20,21]. This may obscure true treatment effects and complicate interpretation of efficacy data. High patient expectations may have contributed to the placebo responses observed in the current study on patient-reported outcomes and other assessments; however, placebo effects were not directly measured, and this remains hypothetical. Although strategies to mitigate placebo responses—such as expectation-neutralizing interventions—have been proposed in recent years, these were not incorporated into the current phase II study design but are to be considered for future research. Additionally, the inclusion of a larger number of study sites and the associated requirement for training in the use of the quantitative TremorTek^®^ device, compared with the pilot trial by Jog et al. [15], may have introduced additional variability.

Formal responder analyses were not performed for the primary outcome or the supporting TETRAS performance, ADL, QUEST, and global impression outcomes. This limits interpretation of whether observed changes were clinically meaningful at the individual-patient level.

In both the current and pilot studies, IncoBoNT-A was well tolerated. In the current phase II trial, completion rates were high (>90%), reflecting sustained patient participation during the approximately 9-month duration study period, as well as favorable trial conduct from the participants’ perspective. No TESAEs were considered related to treatment during either the unilateral or bilateral treatment period. Dynamometer-measured grip strength showed a transient reduction in the incobotulinumtoxinA group and a slight improvement in the placebo group, consistent with the expected local pharmacological effect of BoNT-A on injected forearm muscles. Transient weakness in muscles or muscle groups was anticipated because IncoBoNT-A inhibits acetylcholine release at peripheral cholinergic nerve endings, resulting in a local, reversible reduction in muscle tone.

Muscular weakness was the most commonly reported TEAE during IncoBoNT-A treatment. It occurred more frequently during the placebo-controlled unilateral treatment period (24.0% of patients) than in the pilot study, in which 10.5% of patients receiving customized IncoBoNT-A injections experienced this TEAE [15]. In the current study, muscular weakness events were generally reversible; all events reported in cycle 1 had recovered or resolved by the end of the study, while most events in cycle 2 were either resolved or resolving/recovering at study end. Although IncoBoNT-A treatment was associated with a transient mean reduction in grip strength of the injected UL, muscular weakness did not appear to impact improvement in TETRAS performance dominant UL score or in TETRAS ADL UL scores at week 6 after IncoBoNT-A treatment. Importantly, no cases of severe muscular weakness were observed across either injection Cycle 1 of the current study. Events were generally mild, transient, and localized to the injected UL. These findings are consistent with those of another study using flexible injection protocols of IncoBoNT-A [14] and with results from the recent phase II ELATE trial, in which mild to moderate transient muscular weakness occurred more frequently in the onabotulinumtoxinA group (24.5%) than with placebo (2.5%) when measured using the Tremor Disability Scale-Revised [22]. The ELATE results cited here were reported in a press release and have not yet been published in a peer-reviewed manuscript.

## 4. Conclusions

In conclusion, the primary study endpoint was not met, and the treatment difference in maximum tremor amplitude at the wrist measured with the TremorTek^®^ device numerically favored placebo. Changes in ADL based on TETRAS ADL UL and other clinical measures were observed over time; however, these secondary findings remain exploratory and hypothesis-generating, particularly in the absence of formal responder analyses. IncoBoNT-A administered using the semi-flexible dosing approach was well tolerated with no new or unexpected safety concerns identified, although treatment-related muscular weakness was common and occurred more frequently with IncoBoNT-A than with placebo.

Further research on IncoBoNT-A for the treatment of UL ET is warranted, with careful consideration given to the selection and timing of optimal patient-centric endpoints, such as the TETRAS, and strategies to mitigate placebo response within a multicentric study setting. IncoBoNT-A demonstrated a favorable safety profile without any new or unexpected safety concerns identified.

## 5. Materials and Methods

### 5.1. Study Design and Patients

This was a phase II, prospective, multicenter clinical trial to investigate the efficacy and safety of IncoBoNT-A in patients with ET of the UL (NCT04766723). After an initial randomized, double-blind, placebo-controlled, parallel-group period of unilateral IncoBoNT-A treatment (cycle 1), the study moved to an open-label period of bilateral IncoBoNT-A treatment (cycle 2).

### 5.2. Key Inclusion Criteria

Eligible patients were adults aged ≥18 years with a neurologist-documented diagnosis of ET of at least 3 years duration before enrollment in the study. Patients had isolated tremor syndrome with bilateral UL action tremor, with or without tremor in other locations (e.g., head, voice, or lower limbs), and a score of ≥2 (at least 1 cm tremor amplitude) in at least two of three maneuvers of test item 4 on the TETRAS performance subscale [23,24] as assessed by the investigator and confirmed by an independent TETRAS expert through video assessment. Patients were also required to have the ability to perform all tasks during kinematic tremor assessment and to complete all TETRAS assessments, have visible tremor at wrist level in at least one of the four tasks used in the kinematic tremor assessment of the motor-dominant UL, and fulfill specific TETRAS ADL subscale criteria (i.e., have a score of ≥1 [slight] in at least five of items 2 to 9, a score of ≥3 [moderate] in at least two of the items 2 to 9, and a sum score of ≥9 in items 2 to 9). Eligible patients had body weight ≥50 kg. Stable doses of anti-tremor medication (beta-adrenergic blockers, primidone, gabapentinoids, benzodiazepines, topiramate, all other antiepileptics, centrally acting muscle relaxants) were allowed from at least 4 weeks before screening and throughout the study.

### 5.3. Key Exclusion Criteria

Patients were excluded if they had a history or presence of day-to-day fluctuations in ET that would jeopardize meaningful tremor assessment over time; other neurological signs, such as dystonia, ataxia, or parkinsonism, that could conflict with the ET diagnosis or assessment of ET in ULs; tremor types other than ET; or exposure to tremorgenic drugs within 6 months before the study screening visit or during the study.

### 5.4. Study Design

A schematic of the study design is shown in Figure S1. Following a screening period of up to 3 weeks, eligible patients were randomized (2:1) using an interactive response system to receive double-blind intramuscular injections of IncoBoNT-A or a corresponding volume of placebo, in the muscles of the motor-dominant UL. Randomization was performed in blocks consisting of three randomization numbers. Block-wise distribution of study treatments to study sites was performed centrally to ensure an approximate correct randomization ratio by study site and overall. The block size was not disclosed until the study was unblinded. No other stratification criteria were applied. Doses and muscles to be injected were flexible for forearm and wrist (total forearm and wrist dose 30–65 units), whereas fixed total doses were used for predefined elbow (40 units) and shoulder muscles (60 units). This semi-flexible dosing regimen resulted in an overall total dose per UL ranging from 130 to 165 units (Table S3). Following the baseline visit, patients were monitored at six subsequent visits over a 24-week observation period.

At the end of the unilateral treatment period, patients were assessed for eligibility to participate in the open-label bilateral treatment period. The key criteria for entering the bilateral treatment period were visible tremor of both UL as assessed by the investigator on TETRAS performance subscale item 4, with a score of ≥1.5 in the forward horizontal posture, and/or lateral “wing beating” posture, and/or finger-nose(chin)-finger movement, as well as body weight ≥50 kg. Exclusion criteria for entering the bilateral treatment period were treatment with any other BoNT other than the study medication since the baseline visit and the presence of TESAEs and any infection/inflammation in the planned injection sites.

At the baseline visit for the open-label treatment period, bilateral UL injections with IncoBoNT-A were administered following the same semi-flexible approach to dosing (resulting in a total dose of 260–330 units for both ULs combined). Patients were followed up at three subsequent visits over a 12-week observation period.

Guided injection techniques, including electromyography, electrical stimulation, and/or ultrasound imaging, were employed to identify target muscles and facilitate injection. Although different techniques could be used to identify different muscles within the same patient, the use of at least one guided technique was mandatory for each targeted muscle. The selected technique(s), as determined by the site investigator, were applied consistently across both injection cycles for each patient.

### 5.5. Efficacy Assessments and Endpoints

The primary endpoint was change from study baseline to week 6 in the maximum RMS angular tremor amplitude measurement at wrist level during the unilateral treatment period. Tremor amplitude was assessed in the injected motor-dominant arm at each study visit of cycle 1 with standardized computerized kinematic analysis using a TremorTek^®^ (MDDT Inc., London, ON, Canada [Figure S2]), a device that utilizes wearable movement sensors combined with computer software to determine which muscles and what biomechanics are at play for the patient’s tremor symptoms. Sensors were placed at the shoulder, elbow, forearm, and wrist, and four specific tasks were performed: (1) shoulders flexed at 90° with forearms extended anteriorly and pronated, (2) shoulders flexed at 90° with forearms extended anteriorly in neutral position, (3) holding an empty cup, and (4) holding the same cup filled with 1 pound (0.45 kg) in weight. Amplitude measurements at the wrist, forearm, elbow, and shoulder level across all tasks were conducted as described previously [15] (more details are provided in the Supplementary methods).

TETRAS [23,24] assessments by investigators (performance subscale) and by the patient in the form of an administered questionnaire (ADL subscale) were utilized for derivation of the key secondary endpoints: change from baseline to week 6 in TETRAS performance dominant UL score and in TETRAS ADL UL score in the unilateral period. TETRAS assessments were performed at each study visit. The exact TETRAS scores and items used in this study can be found in Table S2. Other secondary endpoints included change in TETRAS ADL UL Functional Impact score in the unilateral period and change from cycle 2 baseline to week 6 in TETRAS performance dominant UL score, TETRAS performance subscale score, TETRAS ADL UL score, and TETRAS ADL functional impact score in the bilateral period. To our knowledge, no minimal clinically important differences for TETRAS are established.

Patient and investigator GICS for the motor-dominant UL were independently completed by the patient and investigator at the week 6 visit of the unilateral and the bilateral treatment periods (these GICS scores at week 6 of each treatment period were other secondary endpoints).

QUEST, a validated health-related quality-of-life questionnaire for ET [25], was completed by patients at baseline and at week 6 of both treatment periods. This questionnaire has 30 items that inform on interference and impact of ET on quality of life. Changes in QUEST total scores from baseline to week 6 of both treatment cycles were considered other efficacy endpoints. Reductions in QUEST total score indicate improvement in ET-related quality of life. According to Pintér et al. [17], a between-group difference of greater than 4.47 in the QUEST summary index indicates a minimal clinically important difference in health-related quality of life.

### 5.6. Safety Assessments and Endpoints

The secondary safety endpoint for both the unilateral and the bilateral treatment period was the incidence of TEAEs related to treatment as assessed by the investigator. Safety was further assessed by the incidence of all TEAEs, TEAESI, treatment-related TEAESIs, TESAEs, and treatment-related TESAEs during both the unilateral and the bilateral treatment periods. Additional assessments included the investigator’s global assessment of tolerability at the end of the bilateral treatment period and handheld dynamometer maximum grip strength in both hands at the end of the unilateral treatment period.

### 5.7. Statistical Analysis Methods

The results of a previous study [15] suggested that a treatment effect of about one SD could be expected. For a 5% type I error and a randomization ratio of 2:1, a total sample size of 51 analyzable subjects was required to achieve 90% power. To account for uncertainty in the size of the treatment effect and dropouts, it was decided to randomize ~75 patients.

The following analysis sets were defined for the statistical analysis of this study: The safety evaluation set (SES) for each treatment period included all patients who received study treatment during that period. The FAS for the unilateral treatment period was the subset of patients in the respective SES for whom at least one score of tremor amplitude at wrist level (injected motor-dominant UL) at study baseline, and at least one post-baseline score during the unilateral treatment period, were available. The FAS-BP was the subset of patients in the respective SES for whom at least one score of tremor amplitude at wrist level at the open-label treatment period baseline visit, and at least one post-baseline score of both arms during the bilateral treatment period, were available.

All efficacy analyses were based on the FAS for the respective treatment period and safety analyses on the respective SES. Comparisons of treatment groups were performed according to randomized treatment for efficacy endpoints and according to actual treatment for safety endpoints.

The primary analysis of the primary efficacy endpoint was a baseline-adjusted ANCOVA. The difference in LSMs between treatment groups (IncoBoNT-A–placebo) with associated 95% CIs from this model served as an estimate for the size of the treatment effect, with a negative difference indicating a treatment effect in favor of IncoBoNT-A (more details on the primary analysis are provided in the Supplementary methods).

Key secondary efficacy endpoints and secondary and other efficacy endpoints during the unilateral treatment period were analyzed analogously to the primary efficacy endpoint. Secondary efficacy endpoints for the bilateral treatment period, safety endpoints, and other variables were analyzed using standard descriptive statistics. For treatment-related TEAEs, by system organ class and preferred term, occurring in the unilateral placebo-controlled period, risk differences with two-sided 95% CIs were calculated post hoc according to Wilson’s method (Newcombe score [26]). Since this study is powered only for the primary analysis, inferential statistical results for secondary and other endpoints are interpreted descriptively.

### 5.8. Ethics and Informed Consent

The study was conducted in accordance with the ethical principles that have their origin in the Declaration of Helsinki and are consistent with International Council for Harmonization good clinical practice and applicable regulatory requirements. Informed consent was obtained in writing from each patient.

### 5.9. Data Availability

The data presented in this study are available on request from the corresponding author.

## Figures and Tables

**Table 1:** Patient demographics and baseline characteristics of ET ^a^.

**Characteristics evaluated for the SES-UP**	**IncobotulinumtoxinA** **(N = 50)**	**Placebo** **(N = 27)**
Sex		
Female	24 (48.0)	12 (44.4)
Male	26 (52.0)	15 (55.6)
Age, years	64.5 ± 12.7	66.3 ± 9.3
Race ^b^		
White	48 (98.0)	27 (100.0)
Black or African American	1 (2.0)	0 (0.0)
Age of onset of ET, years	38.9 ± 18.7	41.2 ± 19.3
Duration of ET, years	25.6 ± 16.3	25.1 ± 19.5
Family history of ET		
Yes	30 (60.0)	17 (63.0)
No	20 (40.0)	10 (37.0)
Motor-dominant UL		
Left	4 (8.0)	1 (3.7)
Right	46 (92.0)	26 (96.3)
Concomitant diseases	41 (82.0)	21 (77.8)
Concomitant medications (≥1)	44 (88.0)	24 (88.9)
**Characteristics evaluated for the FAS-UP**	**IncobotulinumtoxinA** **(N = 50)**	**Placebo** **(N = 26)**
Maximum tremor amplitude at wrist level of dominant UL [RMS deg]	0.98 ± 1.12	1.16 ± 1.18
TETRAS Performance dominant UL score	13.0 ± 3.0	12.3 ± 2.1
TETRAS ADL UL score	19.4 ± 4.0	19.7 ± 3.0
QUEST total score	33.8 ± 16.2	30.7 ± 15.5

^a^ Data are presented as *n* (%) or mean ± standard deviation. ^b^ Data missing for one patient in the incobotulinumtoxinA group. ADL, activities of daily living; ET, essential tremor; FAS-UP, full analysis set for the unilateral treatment period; QUEST, Quality of life in Essential Tremor; RMS deg, root mean square degrees of arc; SES-UP, safety evaluation set for the unilateral treatment period; TETRAS, The Essential Tremor Rating Assessment Scale; UL, upper limb.

**Table 2:** Summary of treatment-emergent adverse events (TEAEs) in the unilateral treatment period (safety evaluation set for the unilateral treatment period).

TEAEs	IncobotulinumtoxinA (N = 50)	Placebo (N = 27)	Risk Difference [95% Cl]
Any TEAE	30 (60.0)	6 (22.2)	
Any TEAE related to treatment	15 (30.0)	1 (3.7)	
Any TEAE of special interest	13 (26.0)	1 (3.7)	
Any TEAE of special interest related to treatment	12 (24.0)	0 (0.0)	
Any serious TEAE	3 (6.0)	2 (7.4)	
Any serious TEAE related to treatment	0 (0.0)	0 (0.0)	
Any TEAE leading to discontinuation	1 (2.0)	0 (0.0)	
Any TEAE leading to discontinuation related to treatment	0 (0.0)	0 (0.0)	
Any fatal TEAE	0 (0.0)	0 (0.0)	
Any fatal TEAE related to treatment	0 (0.0)	0 (0.0)	
Any TEAE related to COVID-19	0 (0.0)	1 (3.7)	
*TEAEs related to treatment by MedDRA SOC and PT:*			
Musculoskeletal and connective tissue disorders	12 (24.0)	0 (0.0)	24.0 [8.2; 37.4]
Muscular weakness	12 (24.0)	0 (0.0)	24.0 [8.2; 37.4]
General disorders and administration site conditions	3 (6.0)	1 (3.7)	2.3 [−12.8; 13.0]
Injection site hemorrhage	2 (4.0)	1 (3.7)	0.3 [−14.6; 10.2]
Injection site nodule	1 (2.0)	0 (0.0)	2.0 [−10.6; 10.5]

Group-specific data are presented as *n* (%), the risk difference [95% CI] as %. CI, confidence interval; COVID-19, coronavirus disease 2019; MedDRA, Medical Dictionary for Regulatory Activities; PT, preferred term; SOC, system organ class.

**Table 3:** Summary of treatment-emergent adverse events (TEAEs) in the bilateral treatment period (safety evaluation set for the bilateral treatment period).

TEAEs	Total (N = 72)
Any TEAE	29 (40.3)
Any TEAE related to treatment	22 (30.6)
Any TEAE of special interest	19 (26.4)
Any TEAE of special interest related to treatment	19 (26.4)
Any serious TEAE	2 (2.8)
Any serious TEAE related to treatment	0 (0.0)
Any TEAE leading to discontinuation	0 (0.0)
Any TEAE leading to discontinuation related to treatment	0 (0.0)
Any fatal TEAE	0 (0.0)
Any fatal TEAE related to treatment	0 (0.0)
Any TEAE related to COVID-19	1 (1.4)
*TEAEs related to treatment by MedDRA SOC and PT:*	
Musculoskeletal and connective tissue disorders	18 (25.0)
Muscular weakness	17 (23.6)
Muscular fatigue	1 (1.4)
Nervous system disorders	4 (5.6)
Fine motor skill dysfunction	1 (1.4)
Hypoesthesia	1 (1.4)
Monoparesis	1 (1.4)
Paresis	1 (1.4)
General disorders and administration site conditions	2 (2.8)
Injection site pain	2 (2.8)

Data are presented as *n* (%). COVID-19, coronavirus disease 2019; MedDRA, Medical Dictionary for Regulatory Activities; PT, preferred term; SOC, system organ class.

## Data Availability

The data presented in this study are available on request from the corresponding author.

## Supplementary Materials

The following supporting information can be downloaded at: https://www.mdpi.com/article/10.3390/toxins18100435/s1, Supplementary methods: Measurements and primary analysis for the primary efficacy endpoint; Figure S1: Overview of study design; Figure S2: Image of TremorTek^®^ used in the study; Table S1: Time course of change from study baseline to week 24 in maximum grip strength, using hand-held dynamometers, in patients receiving incobotulinumtoxinA or placebo (safety evaluation set for the unilateral treatment period); Table S2: TETRAS subscales and scores used in this study; Table S3: Semi-flexible dosing scheme of incobotulinumtoxinA; Table S4: Extent of exposure during the unilateral treatment period (Cycle 1); Table S5: Extent of exposure during the bilateral treatment period (Cycle 2).

## Abbreviations

The following abbreviations are used in this manuscript:

ADL	Activities of daily living
AE	Adverse event
ANCOVA	Analysis of covariance
BoNT A	Botulinum neurotoxin type A
C2BL	Cycle 2 baseline
CI	Confidence interval
COVID-19	Coronavirus disease 2019
ET	Essential tremor
FAS	Full analysis set
FAS-BP	FAS for the bilateral treatment period
FTM	Fahn-Tolosa-Marin
GICS	global impression of change score
IncoBoNT-A	IncobotulinumtoxinA
LSM	Least squares mean
QUEST	Quality of Life in Essential Tremor
RMS	Root mean square
SD	Standard deviation
SES	Safety evaluation set
TEAE	Treatment-emergent adverse events
TEAESI	TEAEs of special interest
TESAE	Treatment-emergent serious AEs
TETRAS	The Essential Tremor Rating Assessment Scale
UL	Upper limb
