# Supplementary material for: IncobotulinumtoxinA for Upper Limb Essential Tremor: A Randomized, Double-Blind, Placebo-Controlled Trial
Source: Toxins (Basel). 2026 Oct 9;18(10):435. doi: 10.3390/toxins18100435 (PMC13709707; doi:10.3390/toxins18100435)
Supplement: Supplementary file 1 [file toxins-18-00435-s001.zip › toxins-4512866-supplementary.pdf]

Supplementary materials

Supplementary Table S1 Time course of change from study baseline to week 24 in maximum grip strength, using hand-held dynamometers, in patients receiving incobotulinumtoxinA or placebo (safety evaluation set for the unilateral treatment period).

Treatment	Values/Changes (Newton)	Visit	n	Mean	SD
**IncoBoNT-A** (N=50)	Value	Baseline	48	259.8	115.84
	Change from baseline	Week 4	48	–28.8	53.19
		Week 6	48	–24.2	50.68
		Week 8	46	–20.0	48.70
		Week 12	48	–12.5	49.34
		Week 24	45	–8.0	51.02
**Placebo** (N=27)	Value	Baseline	27	262.3	134.81
	Change from baseline	Week 4	27	–5.3	37.81
		Week 6	27	14.6	39.43
		Week 8	27	20.3	36.17
		Week 12	27	15.5	36.86
		Week 24	27	9.4	55.50

IncoBoNT-A, incobotulinumtoxinA; N, number of patients in the safety evaluation set for the unilateral treatment period; n, number of values analyzed; SD, standard deviation.

Supplementary Table S2 TETRAS subscales and scores used in this study.

TETRAS subscales and scores	TETRAS items included	Score range
TETRAS ADL subscale score[a]	All ADL items #1 to #12	0–48
TETRAS ADL UL score	ADL items #2 to #9	0–32
TETRAS ADL Functional Impact score	ADL items #10 to #12	0–12
TETRAS Performance subscale score[b]	All Performance items #1 to #9	0–64
TETRAS Performance dominant UL score	Performance items #4, #6 to #8 of the dominant UL	0–24
TETRAS Performance non-dominant UL score	Performance items #4, #6, and #8 of the non-dominant UL	0–20

[a]Representing the validated TETRAS ADL subscale score with all questionnaire items.
[b]Representing the validated TETRAS Performance subscale score with all test items including tests for both ULs.

ADL, Activities of daily living; TETRAS, The Essential Tremor Rating Assessment Scale; UL, Upper limb.

Supplementary Table S3 Semi-flexible dosing scheme of incobotulinumtoxinA.

Muscles	Number of injection points	Units of incobotulinumtoxinA per muscle	Injection volume per muscle
**Forearm/wrist muscles[a]**	**Total forearm/wrist dose: 30.0–65.0 units**		
M. extensor carpi ulnaris	1	2.5–5.0 units	0.025–0.05 mL
M. extensor carpi radialis	1	2.5–5.0 units	0.025–0.05 mL
M. flexor carpi radialis	1	5.0–15.0 units	0.05–0.15 mL
M. flexor carpi ulnaris	1	5.0–15.0 units	0.05–0.15 mL
M. pronator teres	1	5.0–15.0 units	0.05–0.15 mL
M. pronator quadratus	1	2.5–5.0 units	0.025–0.05 mL
M. supinator	1	2.5–5.0 units	0.025–0.05 mL
**Elbow muscles**	**Elbow total dose: 40.0 units**		
M. brachialis	2	20.0 units	0.20 mL
M. triceps brachii	2	20.0 units	0.20 mL
**Shoulder muscles**	**Shoulder total dose: 60.0 units**		
M. latissimus dorsi	2	15.0 units	0.15 mL
M. pectoralis major	2	15.0 units	0.15 mL
M. supraspinatus	3	15.0 units	0.15 mL
M. infraspinatus	3	15.0 units	0.15 mL
**Total dose per upper limb**		**130.0–165.0 units**	**1.30–1.65 mL**

[a] At least four of the listed seven forearm/wrist muscles had to be injected.

M, musculus.

Supplementary Table S4 Extent of exposure during the unilateral treatment period (Cycle 1).

Muscle group	Muscle	IncobotulinumtoxinA (N = 50) n	%	Mean	SD	Placebo (N = 27) n	%	Mean	SD
Total	Total	50	100.0%	1.47	0.182	27	100.0%	1.51	0.134
Forearm/wrist	Total	50	100.0%	0.48	0.132	27	100.0%	0.51	0.134
	M. extensor carpi ulnaris	47	94.0%	0.05	0.013	25	92.6%	0.05	0.013
	M. extensor carpi radialis	46	92.0%	0.05	0.013	25	92.6%	0.05	0.013
	M. flexor carpi radialis	50	100.0%	0.12	0.035	27	100.0%	0.13	0.031
	M. flexor carpi ulnaris	50	100.0%	0.12	0.035	27	100.0%	0.13	0.025
	M. pronator teres	45	90.0%	0.11	0.043	24	88.9%	0.11	0.045
	M. pronator quadratus	31	62.0%	0.05	0.008	17	63.0%	0.05	0.014
	M. supinator	29	58.0%	0.05	0.028	16	59.3%	0.05	0.006
Elbow	Total	50	100.0%	0.39	0.042	27	100.0%	0.40	0.000
	M. brachialis	50	100.0%	0.20	0.014	27	100.0%	0.20	0.000
	M. triceps brachii	49	98.0%	0.20	0.000	27	100.0%	0.20	0.000
Shoulder	Total	49	98.0%	0.60	0.000	27	100.0%	0.60	0.000
	M. latissimus dorsi	49	98.0%	0.15	0.000	27	100.0%	0.15	0.000
	M. pectoralis major	49	98.0%	0.15	0.000	27	100.0%	0.15	0.000
	M. supraspinatus	49	98.0%	0.15	0.000	27	100.0%	0.15	0.000
	M. infraspinatus	49	98.0%	0.15	0.000	27	100.0%	0.15	0.000

For incobotulinumtoxinA-treated patients, an injection volume of 0.1 mL corresponds to a dose of 1 U incobotulinumtoxinA.

M, musculus; N, number of patients in the safety evaluation set for the unilateral treatment period; n, number of patients injected in respective muscle/muscle group in the safety evaluation set for the unilateral treatment period (by treatment); SD, standard deviation.

Supplementary Table S5 Extent of exposure during the bilateral treatment period (Cycle 2).

Muscle group	Muscle	Dominant UL				Non-dominant UL			
		n	%	Mean	SD	n	%	Mean	SD
Total	Total	72	100.0%	1.47	0.138	72	100.0%	1.46	0.142
Forearm/wrist	Total	72	100.0%	0.47	0.139	72	100.0%	0.47	0.144
	M. extensor carpi ulnaris	67	93.1%	0.05	0.010	67	93.1%	0.04	0.010
	M. extensor carpi radialis	67	93.1%	0.05	0.012	67	93.1%	0.04	0.010
	M. flexor carpi radialis	71	98.6%	0.12	0.034	72	100.0%	0.12	0.036
	M. flexor carpi ulnaris	70	97.2%	0.12	0.037	69	95.8%	0.12	0.039
	M. pronator teres	66	91.7%	0.11	0.044	66	91.7%	0.11	0.044
	M. pronator quadratus	42	58.3%	0.05	0.008	42	58.3%	0.05	0.008
	M. supinator	42	58.3%	0.05	0.008	41	56.9%	0.05	0.009
Elbow	Total	72	100.0%	0.40	0.012	72	100.0%	0.40	0.024
	M. brachialis	72	100.0%	0.20	0.000	72	100.0%	0.20	0.012
	M. triceps brachii	72	100.0%	0.20	0.012	72	100.0%	0.20	0.012
Shoulder	Total	72	100.0%	0.60	0.000	72	100.0%	0.60	0.006
	M. latissimus dorsi	72	100.0%	0.15	0.000	72	100.0%	0.15	0.000
	M. pectoralis major	72	100.0%	0.15	0.000	72	100.0%	0.15	0.006
	M. supraspinatus	72	100.0%	0.15	0.000	72	100.0%	0.15	0.000
	M. infraspinatus	72	100.0%	0.15	0.000	72	100.0%	0.15	0.000

For incobotulinumtoxinA-treated patients, an injection volume of 0.1 mL corresponds to a dose of 1 U incobotulinumtoxinA.

M, musculus; n, number of patients injected in respective muscle/muscle group in the safety evaluation set for the bilateral treatment period (by UL); SD, standard deviation; UL, upper limb.

Supplementary Figure S1 Overview of study design.

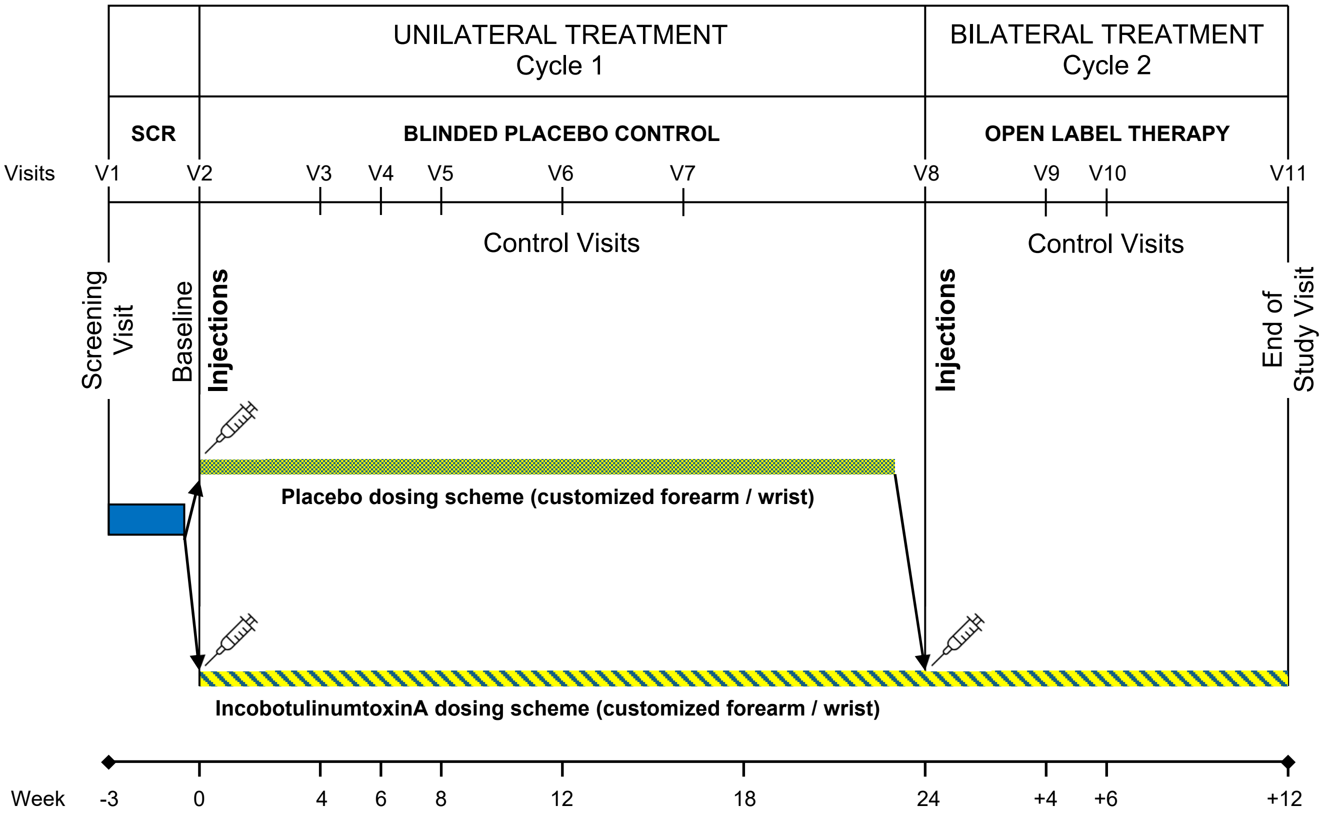

Patients were randomized at a ratio of 2:1 to receive incobotulinumtoxinA or matching placebo. Patients received injections of incobotulinumtoxinA or matching placebo into the motor-dominant upper limb on day 1 of the unilateral treatment period (Cycle 1), followed by a 24-week observation period. On day 1 of the bilateral treatment period (Cycle 2; V8), patients received injections of incobotulinumtoxinA into both arms followed by a 12-week observation period. The overall study duration was 36 weeks plus an up to 3-week screening period.

SCR, screening; V, visit.

Supplementary Figure S2 Image of TremorTek® used in the study.

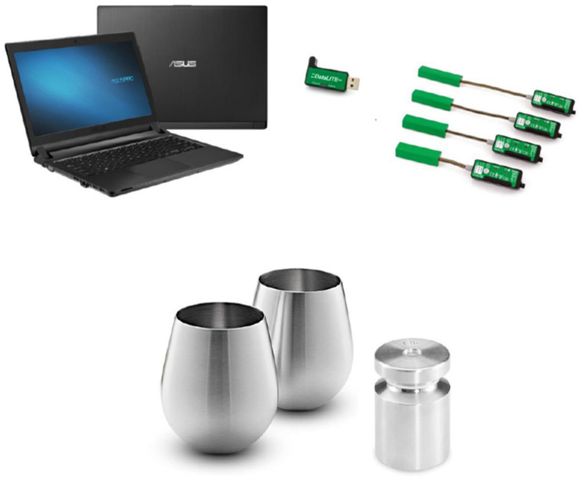

Supplementary methods

Primary analysis of the primary efficacy endpoint

The primary analysis of the primary efficacy endpoint was based on the full analysis set for the unilateral period (observed cases) using least squares means (LSMs) from a baseline-adjusted analysis of covariance as implemented in SAS (v9.4) PROC MIXED. Fixed class effects were the treatment group (incobotulinumtoxinA versus placebo) and study site. Study baseline angular tremor amplitude at wrist level for the task with the maximum amplitude was the fixed continuous covariate. Variances were allowed to differ between treatment groups. Denominator degrees of freedom were calculated according to the Kenward–Roger method (Kenward and Roger, 1997). The difference in LSMs between treatment groups with associated 95% confidence intervals from this model served as an estimate for the size of the treatment effect. The two-sided t-test of the LSM treatment difference was applied to the following testing problem at $\alpha_{2\text{-sided}}$ = 5%:

H0: $\mu_{NT201} - \mu_{Placebo} = 0$ vs. H1: $\mu_{NT201} - \mu_{Placebo} \neq 0$,

where $\mu_{NT201} - \mu_{Placebo}$ denotes the expected mean treatment difference on the primary efficacy endpoint, with a negative difference indicating a treatment effect in favor of incobotulinumtoxinA.
